# Capacity and Entropy of a Retro-Causal Channel Observed in a Twin Mach-Zehnder Interferometer During Measurements of Pre- and Post-Selected Quantum Systems

**DOI:** 10.3390/e20060411

**Published:** 2018-05-27

**Authors:** Allen Parks, Scott Spence

**Affiliations:** Electromagnetic and Sensor Systems Department, Naval Surface Warfare Center Dahlgren Division, Dahlgren, VA 22448, USA

**Keywords:** quantum measurement, pre- and post-selected systems, retro-causal channel, channel capacity, channel entropy

## Abstract

Simple intuitive models are presented for the capacity and entropy of retro-causal channels in measured ensembles of quantum systems which can be represented as statistical mixtures of pre-selected only and pre- and post-selected systems. Measurement data from a twin Mach-Zehnder interferometer experiment are used in these models to discuss the capacity and entropy of an apparent retro-causal channel observed in the experimental data. It is noted that low capacity/low entropy retro-causal channels can exist in strong measurement systems.

## 1. Introduction

The possibility that future events can influence the present has long been argued by physicists and philosophers. In 1964 Aharonov, Bergmann, and Lebowitz proposed a time symmetric theory for non-relativistic quantum mechanics [1] and was further developed by Aharonov et al. in terms of weak measurements and weak values [2,3]. This theory not only employs standard forward in time evolving quantum states, but also the retro-causal property of quantum states evolving backward in time. Although the retro-causal interpretation of weak value theory is controversial, e.g., [4,5,6,7], experiments performed in recent years have verified many of the theory’s counterintuitive predictions, e.g., [8,9,10,11,12].

Inspired by a weak value gedanken experiment discussed by Tollaksen et al. [13], an optical twin Mach-Zehnder interferometer (MZI) experiment was performed in 2010 which confirmed the predictions made by the gedanken experiment and provided indirect experimental evidence that single particle quantum interference phenomena can be explained in terms of a non-local exchange of modular momentum [14,15]. A recent re-analysis of the reduced 2010 experimental data also suggests that a possible retro-causal channel was observed in the twin MZI apparatus during the associated measurements of ensembles of pre-selected and post-selected (PPS) quantum systems [16,17].

Using the data from the 2010 experiment as a guide, simple models are presented here for the capacity and entropy of this retro-causal channel. The capacity model assumes that the probability distribution for the measurement pointer can be represented as a statistical mixture of the probability distributions for the pointers associated with the PPS systems and the pre-selected only (PSO) systems produced by the measurement. The entropy of the channel is modelled as a classical binary entropy function for a Bernoulli process. Application of the capacity model to the 2010 experimental data shows that even though the capacity of the channel is greatest when the measurement is weak, the channel persists—albeit with increasingly small capacity—as the measurement becomes stronger. It is also shown that—as expected for a binary entropy function—the entropy is smallest when the measurement is extremely weak or extremely strong and reaches its maximum value when the pointer distribution of the mixture is the mean of the PPS and PSO pointer distributions.

An overview of the theory of projector measurement for PPS and PSO systems is given in Section 2 (measurements of a projector were made in the 2010 experiment). The capacity and entropy models for retro-causal channels present in PPS and PSO mixtures (like that assumed for the 2010 experiment) are presented in Section 3. Section 4 reviews the relevant features of the 2010 experiment and discusses—from the perspective of these models—the properties of the retro-causal channel associated with the twin MZI used in the experiment. Concluding remarks comprise the final section of this paper.

## 2. Theory of Projector Measurement

The measured value of a quantum mechanical observable for a PSO (PPS) system is the statistical result of a standard measurement procedure performed upon an ensemble of identical PSO (PPS) quantum systems. Such measurements can be described using the von Neumann description of a quantum measurement at time $t_{0}$ of a time independent observable A that describes a quantum system in an initial fixed pre-selected state $|\psi_{i}\rangle =\sum_{J}c_{j}|a_{j}\rangle$ at $t_{0}$, where the set J indexes the eigenstates $|a_{j}\rangle$ of the operator $\hat{A}$. In this description, the interaction between the measurement apparatus—i.e., the pointer—and the quantum system is described by the von Neumann interaction operator $\hat{V}$ given by:

$$\hat{V}=e^{-\frac{i}{\hbar }\int \hat{H}dt}=e^{-\frac{i}{\hbar }\gamma \hat{A}\hat{p}},$$

where $\gamma =\int \gamma \delta \left(t-t_{0}\right)dt$ defines the strength of the measurement’s impulsive interaction at $t_{0}$ and $\hat{p}$ is the momentum operator for the pointer of the measurement apparatus, which is in the initial state |ϕ〉. Let $\hat{q}$ be the pointer’s position operator that is conjugate to $\hat{p}$ and assume that ϕ(q)≡〈q|ϕ〉 is real valued. When the observable A to be measured is a projector—as was the case in the 2010 experiment—the interaction operator is given exactly by [18]:

$$\hat{V}=\hat{1}-\hat{A}+\hat{A}\hat{T},$$

where $\hat{T}=e^{-\frac{i}{\hbar }\gamma \hat{p}}$ is the spatial translation operator defined by the action $\langle q|\hat{T}|\phi \rangle =\phi \left(q-\gamma \right)$.

Prior to the measurement of projector $\hat{A}$, the pre-selected system and the pointer are in the tensor product state $|\psi_{i}\rangle |\phi \rangle$. Immediately after the measurement, the combined system is in the PSO pointer state given—for arbitrary interaction strength—exactly by:

(1) $$|\Phi \rangle =\hat{V}|\psi_{i}\rangle |\phi \rangle =\left(\hat{1}-\hat{A}+\hat{A}\hat{T}\right)|\psi_{i}\rangle |\phi \rangle$$

and which yields:

(2) $$\langle \Phi |\hat{q}|\Phi \rangle =\langle \phi |\hat{q}|\phi \rangle +\gamma \langle \psi_{i}|\hat{A}|\psi_{i}\rangle$$

and:

(3) $${\left|\langle q|\Phi \rangle \right|}^{2}=\left(1-\langle \psi_{i}|\hat{A}|\psi_{i}\rangle \right){\left|\langle q|\phi \rangle \right|}^{2}+\langle \psi_{i}|\hat{A}|\psi_{i}\rangle {\left|\langle q|\hat{T}|\phi \rangle \right|}^{2}$$

as the exact mean PSO pointer position and pointer distribution profile, respectively. Note that since there are no interference cross terms in Equation (3), the profile ${\left|\langle q|\Phi \rangle \right|}^{2}$ does not exhibit interference.

If the state $|\psi_{f}\rangle$, $\langle \psi_{f}|\psi_{i}\rangle \neq 0$, is post-selected at $t_{0}$, the resulting expression for the PPS pointer state is given—regardless of interaction strength—exactly by:

(4) $$|\Psi \rangle =\langle \psi_{f}|\Phi \rangle =\frac{e^{i\chi }}{N}\left[\left(1-A_{w}\right)\hat{1}+A_{w}\hat{T}\right]|\phi \rangle ,$$

where:

$$A_{w}\equiv \frac{\langle \psi_{f}|\hat{A}|\psi_{i}\rangle }{\langle \psi_{f}|\psi_{i}\rangle }\equiv {\left(A^{1}\right)}_{w}$$

is the complex valued *weak value* of A (e.g., [2,3]); χ is the Pancharatnam phase defined by:

$$e^{i\chi }=\frac{\langle \psi_{f}|\psi_{i}\rangle }{\left|\langle \psi_{f}|\psi_{i}\rangle \right|};$$

and:

$$N=\sqrt{a+J\left(\hat{1}\right)}$$

is the normalization factor. Here:

$$a=1-2ReA_{w}+2{\left|A_{w}\right|}^{2}$$

and for any Hermitean $\hat{x}$:

$$J\left(\hat{x}\right)=A_{w}\left(1-A_{w}^{*}\right)\langle \phi |\hat{x}\hat{T}|\phi \rangle +A_{w}^{*}\left(1-A_{w}\right)\langle \phi |{\hat{T}}^{†}\hat{x}|\phi \rangle .$$

The associated mean PPS pointer position and pointer distribution profile are given exactly by:

(5) $$\langle \Psi |\hat{q}|\Psi \rangle =\frac{1}{N^{2}}\left[a\langle \phi |\hat{q}|\phi \rangle +J\left(\hat{q}\right)+\gamma {\left|A_{w}\right|}^{2}\right]$$

and:

(6) $${\left|\langle q|\Psi \rangle \right|}^{2}=\left(\frac{1}{N^{2}}\right)\left\{\begin{array}{c} {\left|1-A_{w}\right|}^{2}{\left|\langle q|\phi \rangle \right|}^{2}+{\left|A_{w}\right|}^{2}{\left|\langle q|\hat{T}|\phi \rangle \right|}^{2}\\ +2Re\left[A_{w}\left(1-A_{w}^{*}\right){\langle q|\phi \rangle }^{*}\langle q|\hat{T}|\phi \rangle \right] \end{array}\right\}$$

Note that since Equation (6) contains interference cross terms, the profile ${\left|\langle q|\Psi \rangle \right|}^{2}$ exhibits interference. Of course, the PPS states are selected at times $t_{i}<t_{0}<t_{f}$ and must be evolved forward and backward in time, respectively, to the measurement time $t_{0}$. It is important to note here that such PPS systems imply retro-causality, since the weak value of A is measured and $|\psi_{f}\rangle$ is the post-selected state at measurement time $t_{0}<t_{f}$.

A weak measurement of A occurs when the interaction strength γ is sufficiently small so that the system is essentially undisturbed by the measurement and the pointer’s position uncertainty Δq is much larger than the separation between $\hat{A}$’s eigenvalues (if a PPS system undergoes a weak measurement of A, then the resulting value of A is its weak value $A_{w}$). In order for measurements to qualify as weak measurements, the associated momentum uncertainty of the pointer must simultaneously satisfy the following two formal weakness conditions which define the extreme upper bound $\gamma_{w}$ for the weak measurement regime, e.g., [14,19]:

$$\Delta p\ll \frac{\hbar }{\gamma }{\left|A_{w}\right|}^{-1}$$

and

$$\Delta p\ll \underset{\left(n=2,3,\cdots \right)}{\min}\frac{\hbar }{\gamma }{\left|\frac{A_{w}}{{\left(A^{n}\right)}_{w}}\right|}^{1/\left(n-1\right)}.$$

A measurement performed in accordance with these inequalities such that $\gamma \ll \gamma_{w}$ is a weak measurement whereas a measurement performed with a sufficiently large $\gamma \gg \gamma_{w}$ is a strong measurement. A measurement which is neither weak nor strong is a transition measurement, i.e., it is a measurement performed in the transition region between a weak measurement and a strong measurement. Although the measurements in the 2010 experiment were made—not only in the weak measurement regime—but also in the transition and strong measurement regions, Equations (1) and (4) still apply over this range of interaction strengths since these expressions are valid regardless of the interaction strength.

## 3. Capacity and Entropy Models for Retro-Causal Channels Present in PPS and PSO Mixtures

Suppose a projector measurement of fixed interaction strength of an ensemble of quantum systems produces independent measurement pointer distributions for both PPS and PSO systems (i.e., there is no phase relationship between the PPS and PSO states) such that the associated distribution profile ${\left|\langle q|\Lambda \rangle \right|}^{2}$ for the measurement pointer can be modelled as the statistical mixture

(7) $${\left|\langle q|\Lambda \rangle \right|}^{2}=\alpha {\left|\langle q|\Psi \rangle \right|}^{2}+\beta {\left|\langle q|\Phi \rangle \right|}^{2}.$$

Here ${\left|\langle q|\Psi \rangle \right|}^{2}$ and ${\left|\langle q|\Phi \rangle \right|}^{2}$ are the normalized distributions for the PPS and PSO measurement pointers given by Equations (6) and (3), respectively, and 0≤α≤1 (β=1−α) is the fraction of PPS (PSO) systems produced by the measurement. Clearly, if α=1 (β=0), the measured ensemble is comprised only of PPS systems, whereas if β=1 (α=0), the ensemble is comprised entirely of PSO systems.

The model also assumes that: (i) only the measured presence of weak values in PPS systems induces retro-causal channels which permit the backward in time evolution of post-selected states from $t_{f}$ to $t_{0}$; and (ii) since the weak value measurement of PPS systems implies retro-causality, the presence of such PPS systems in a mixture indicates the presence of a retro-causal channel during a measurement process. Based upon these assumptions and Equation (7), the capacity C for the retro-causal channel in a statistical mixture of PPS and PSO systems can be defined as the fraction of the mixture that is comprised of PPS systems, i.e.,

C≡α.

When the fraction α is unknown, then C can be determined from knowledge of the associated mean pointer positions. To see this, observe that Equation (7) can be used to relate the mean pointer position for a mixture to the mean pointer positions for the PPS and PSO constituents of the mixture. In particular:

$$\int q{\left|\langle q|\Lambda \rangle \right|}^{2}dq=\alpha \int q{\left|\langle q|\Psi \rangle \right|}^{2}dq+\beta \int q{\left|\langle q|\Phi \rangle \right|}^{2}dq.$$

After identifying each integral in the last equation with the appropriate mean pointer position and setting α=C and β=1−C, the expression:

$$\langle \Lambda |\hat{q}|\Lambda \rangle =\alpha \langle \Psi |\hat{q}|\Psi \rangle +\beta \langle \Phi |\hat{q}|\Phi \rangle =\langle \Phi |\hat{q}|\Phi \rangle +C\left(\langle \Psi |\hat{q}|\Psi \rangle -\langle \Phi |\hat{q}|\Phi \rangle \right)$$

is obtained which can be readily solved for C to yield:

(8) $$C=\frac{\langle \Lambda |\hat{q}|\Lambda \rangle -\langle \Phi |\hat{q}|\Phi \rangle }{\langle \Psi |\hat{q}|\Psi \rangle -\langle \Phi |\hat{q}|\Phi \rangle },\;\langle \Psi |\hat{q}|\Psi \rangle \neq \langle \Phi |\hat{q}|\Phi \rangle .$$

Here $\langle \Lambda |\hat{q}|\Lambda \rangle ,\langle \Phi |\hat{q}|\Phi \rangle ,$ and $\langle \Psi |\hat{q}|\Psi \rangle$ are the mean pointer positions for the mixture, the PSO systems in the mixture (Equation (2)), and the PPS systems in the mixture (Equation (5)), respectively. It is important to note that Equation (8) cannot be used to determine C when the associated PPS and PSO pointer positions are equal (since C is undefined) or when the pointer positions are such that C<0 (since 0≤C≤1). Also, observe that Equation (8) has the following requisite properties: (i) if C=1, then $\langle \Lambda |\hat{q}|\Lambda \rangle =\langle \Psi |\hat{q}|\Psi \rangle$; and (ii) if C=0, then $\langle \Lambda |\hat{q}|\Lambda \rangle =\langle \Phi |\hat{q}|\Phi \rangle$.

In order to associate an entropy with such a retro-causal channel it is further assumed that—for a fixed interaction strength and a fixed C—a measured system can be treated as a random variable in a Bernoulli process such that after the measurement it is either a PPS system with probability C or a PSO system with probability 1−C. The classical entropy H of the channel (in Shannons) is then defined here as the binary entropy function for the Bernoulli process given by:

(9) $$H=-C\log_{2}C-\left(1-C\right)\log_{2}\left(1-C\right).$$

Relying upon the standard interpretation of a binary entropy function, 0≤H≤1 can be viewed as a measure of the uncertainty associated with a measurement outcome: when C=1 (0), then it is certain that the measurement produces a PPS (PSO) system and H=0; maximum uncertainty is achieved when $C=\frac{1}{2}$ in which case H=1. It is easy to see from Equations (7) and (8) that—as anticipated—maximum uncertainty is achieved when:

$${\left|\langle q|\Lambda \rangle \right|}^{2}=\frac{1}{2}\left({\left|\langle q|\Psi \rangle \right|}^{2}+{\left|\langle q|\Phi \rangle \right|}^{2}\right)$$

or when:

$$\langle \Lambda |\hat{q}|\Lambda \rangle =\frac{1}{2}\left(\langle \Psi |\hat{q}|\Psi \rangle +\langle \Phi |\hat{q}|\Phi \rangle \right)$$

## 4. The Retro-Causal Channel in the 2010 Twin Mach-Zehnder Interferometer Experiment

Now consider the 2010 twin Mach-Zehnder experiment mentioned above [14,16]. In that experiment, the mean pointer position measurements observed at the output port of the third beamsplitter were essentially derived from the overlapping at the second beamsplitter BS2 of the two beams traversing the arms in the interferometer between the first and second beamsplitters. When γ=0, i.e., there is no measurement—the two beams completely overlap at BS2. However, as γ increases the beam overlap at BS2 decreases and the non-overlap region of the beams on BS2 increases.

To apply the channel capacity model to the experimental data note that—from an operational perspective—interference only occurs in the overlap region (at BS2) and that—from a theoretical perspective—only the PPS distribution given by Equation (6) exhibits interference. Pursuant to assumptions (i) and (ii) in the last section, the beam overlap region corresponds to the retro-causal channel in the apparatus. By similar reasoning, since interference does not occur in the non-overlap region and only the PSO distribution given by Equation (3) exhibits no interference, then the non-overlap region does not correspond to a retro-causal channel (operationally, the second MZI in the apparatus effectively responds to photons in the non-overlap region as though they never traversed the first MZI and enter as such the input ports of the second MZI as PSO systems). Consequently, the measured pointer distribution can be modelled as a mixture of independent PSO and PPS pointer distributions. It follows that as the beam overlap decreases, ${\left|\langle q|\Phi \rangle \right|}^{2}$ becomes increasingly dominant in the mixture and $\langle \Lambda |\hat{q}|\Lambda \rangle$ is increasingly dominated by $\langle \Phi |\hat{q}|\Phi \rangle$.

Here such measurements are viewed from a simplified perspective as a classical Bernoulli process which “sorts” measured systems into PSO and PPS “bins”: measured systems which do not “intercept” post-selected states at the time of measurement go into the PSO bin, whereas those that do go into the PPS bin. As γ increases, the number of systems occupying the PPS bin (i.e., the capacity C) decreases.

Although the channel capacity is not directly measured in this experiment, it can be indirectly estimated using Equation (8) and the measurement pointer data presented in Figure 1 in [18] for the case $A_{w}=1$. In that figure the vertical axis corresponds to pointer position in μm referenced to $\langle \phi |\hat{q}|\phi \rangle =0$ and the x values along the horizontal axis correspond to the interaction strengths γ=−1.5x μm. The lower curve in the figure labeled “statistical mixture $A_{w}=1$” corresponds to the pointer positions $\langle \Lambda |\hat{q}|\Lambda \rangle$, the line labeled “PSO theoretical 〈A〉=½” corresponds to $\langle \Phi |\hat{q}|\Phi \rangle$, and the line labeled “PPS theoretical no ‘collapse’ $A_{w}=1$” corresponds to $\langle \Psi |\hat{q}|\Psi \rangle$. Referring to the figure, if x=300 μm (i.e., γ=−450 μm and the measurement is a transition measurement), then $\langle \Lambda |\hat{q}|\Lambda \rangle ≅-325\;\mu m$, $\langle \Phi |\hat{q}|\Phi \rangle ≅-225\;\mu m$, and $\langle \Psi |\hat{q}|\Psi \rangle ≅-450\;\mu m$. Substituting these values into Equation (8) yields C≅0.444 as the empirical estimate for the capacity of the associated retro-causal channel and corresponds to the fraction of PPS systems in the mixture when γ=−450 μm. The classical entropy of the channel can also be estimated using this value for the capacity in Equation (9) to obtain $H≅-0.444\;\log_{2}\left(0.444\right)-0.556\log_{2}\left(0.556\right)=0.991$. Thus, when γ=−450 μm the uncertainty is nearly maximum as to whether the outcome of a measurement will be a PSO system or a PPS system.

Also, observe from Figure 1 that as x (i.e., γ) increases and the measurement becomes stronger, the pointer position $\langle \Lambda |\hat{q}|\Lambda \rangle$ for the mixture converges towards the pointer position $\langle \Phi |\hat{q}|\Phi \rangle$ for PSO systems, whereas the pointer position $\langle \Psi |\hat{q}|\Psi \rangle$ for PPS systems diverges from $\langle \Phi |\hat{q}|\Phi \rangle$. Application of these trends to Equations (8) and (9) shows the expected behavior that as the interaction strength increases and approaches that of a strong measurement, both the channel capacity and entropy approach zero. Conversely, as x approaches 0, $\langle \Lambda |\hat{q}|\Lambda \rangle$ converges towards $\langle \Psi |\hat{q}|\Psi \rangle$ while both $\langle \Psi |\hat{q}|\Psi \rangle$ and $\langle \Phi |\hat{q}|\Phi \rangle$ approach one another at x=0. Using these trends in Equations (8) and (9) again shows the expected behavior, that as the measurement becomes weak, the capacity approaches unity and the entropy vanishes.

## 5. Discussion

Although the models presented here are simple, they provide an intuitive description of the capacity and entropy of the apparent retro-causal channel in the 2010 experimental data. This includes the perhaps unexpected possibility that non-vanishing retro-causal channels persist in weak value-measured PPS ensembles even when the measurements are strong (e.g., if the two apparently non-overlapping beams resulting from a strong measurement have Gaussian distributions—as was the case for the 2010 experiment—the associated capacity theoretically approaches zero asymptotically as γ→∞ since the wings of these distributions still overlap).

Regardless of the fact that the model assumes the use of a projector measurement (because the 2010 experiment involved projector measurements and the presence (absence) of interference in the associated pointer theories provide the basis for assigning PPS (PSO) systems to the overlap (non-overlap) region), the capacity model should be applicable for non-projector measurements of ensembles which have pointer probability distribution functions that can be reasonably represented as a statistical mixture of a weak value measured PPS pointer distribution function and a PSO pointer distribution function. The model is not valid for situations which do not possess an associated weak value measured PPS pointer distribution because assumptions (i) and (ii) in Section 3 are violated. As implied in Section 3, the utility of the model is also limited by the fact that Equation (8) can only be used to estimate the capacity when values for the mean pointer distributions for the mixture and the PPS and PSO components are known.

Before closing, it is noted that although the pointer distributions associated with the capacity model are quantum mechanical, the entropy H used here is effectively the classical information theoretic Shannon entropy and was selected for its simple adequate description of the measurement process as a series of Bernoulli trials. Although the Shannon entropy has a natural extension to quantum systems via the von Neumann entropy $S=-Tr\left\{\hat{\rho }\log\hat{\rho }\right\}$, where $\hat{\rho }$ is the statistical operator for the system, H was chosen for use here instead of its quantum mechanical counterpart to avoid introducing unnecessary complexity into describing the measurement process, e.g., [20].
